# Meniscus surgery is still widely performed in the treatment of degenerative meniscus tears in The Netherlands

**DOI:** 10.1007/s00167-017-4473-2

**Published:** 2017-03-03

**Authors:** Jan J. Rongen, Tony G. van Tienen, Pieter Buma, Gerjon Hannink

**Affiliations:** 1 Department of operating rooms, Radboud University Medical Center, Radboud Institute for Health Sciences, PO Box 9101, 6500 HB Nijmegen, The Netherlands; 2 Kliniek Viasana, Mill, The Netherlands; 3 Orthopaedic Research Laboratory, Radboud University Medical Center, Radboud Institute for Molecular Life Sciences, PO Box 9101, 6500 HB Nijmegen, The Netherlands

**Keywords:** Incidences, Meniscus: arthroscopy, Meniscectomy, Meniscal, Registry, Guidelines

## Abstract

**Purpose:**

Studies have demonstrated rising incidences of meniscus procedures for degenerative meniscus tears in several countries, despite accumulating evidence that questions the efficacy of the treatment. It is not clear if this rise in incidences also applies to the practice of arthroscopic surgery in the Netherlands. The objective of this study was, therefore, to evaluate the number of meniscal surgeries performed in the Netherlands between 2005 and 2014.

**Methods:**

We used registry-based data on meniscal surgeries that originated from Dutch national hospital basic care registrations from 2005 to 2014. Poisson regression models were used to test differences in incidences of meniscus surgeries performed in the Netherlands between 2005 and 2014, and to find out if changes in incidences over this period differed for younger and older patients.

**Results:**

The number of meniscus surgeries was highest in patients aged 40–65 years, who accounted for half of the total number of meniscal surgeries. The incidences of meniscus surgeries decreased from 2005 to 2014 (*p* < 0.001); this decrease was observed in all age groups, although the decrease in incidences was more pronounced for younger patients (aged less than 40 years) compared to middle-aged and older patients (aged 40 years and older) (*p* < 0.001).

**Conclusions:**

The implementation of a nationwide guideline for arthroscopic procedures for meniscus tears may have contributed to a decrease in incidences of meniscus procedures. Despite accumulating evidence that questions the rationalisation and effectiveness of the treatment, meniscus surgery is still widely performed in the treatment of degenerative meniscus tears in the Netherlands, demonstrating a delay in the dissemination, acceptance, and implementation of clinical evidence in the practice of arthroscopic surgery in the Netherlands.

**Level of evidence:**

II.

## Introduction

Arthroscopic knee surgery with meniscectomy is a well-established surgical procedure in the treatment of symptoms attributed to degenerative meniscus tears [1–3]. Degenerative meniscus tears are typically seen in middle-aged and older patients in knees that have already demonstrated signs of knee osteoarthritis and are caused by chronic degenerative processes [4, 5].

Clinical symptoms routinely attributed to degenerative meniscus tears generally have a gradual onset and encompass localised knee pain and mechanical symptoms, such as occasional catching or locking of the knee [6–8]. Common rationalisation for arthroscopic surgery is that the clinical symptoms are attributable to a mechanical problem, and that the degenerative meniscus tear is the cause of this mechanical problem [6].

However, over the past decade, evidence has accumulated that questions both the rationalisation and the effectiveness of arthroscopic surgery for degenerative meniscus tears.

First, it has been demonstrated that the sensitivity and specificity of the symptoms attributed to the degenerative meniscus tear in the osteoarthritic knee are low [9–12]. Moreover, asymptomatic meniscus tears are highly prevalent amongst people with knee osteoarthritis [13–15]. These observations cast doubt on the rationalisation for the arthroscopic treatment of degenerative meniscus tears.

Second, randomised controlled trials have demonstrated that the treatment effect, if present at all, is inconsequential, for a part attributable to a placebo effect, and does not outweigh the short-term harm [7, 16–20].

Based on this accumulating evidence, a decrease in incidence of meniscus surgeries would be expected. In contrast, however, studies have demonstrated rising incidences of meniscus surgeries for degenerative meniscus tears across several countries [1, 21, 27]. This observation demonstrates a delay in the dissemination, acceptance and implementation of clinical evidence into orthopaedic practice. However, it is not clear if this observation also applies to the practice of arthroscopic surgery in the Netherlands since an updated nationwide guideline for arthroscopic procedures was introduced in 2010. In brief, this guideline introduced the use of MRI in the diagnostic process of meniscus tears in younger patients, and advised surgeons to be cautious about the surgical treatment of degenerative meniscus tears [22]. In preparation of the guideline, all members of the Netherlands Orthopaedic Association were given the opportunity to comment on its content, and the final guideline version was approved during the Annual General Meeting of the Netherlands Orthopaedic Association. Moreover, an accredited Continuing Medication Education (CME) that addressed the guideline content was made available to all orthopaedic surgeons. The implementation of the guideline may have led to a different trend in meniscus surgeries, as compared to rising incidences in other countries. The objective of this study was, therefore, to evaluate the number of meniscus surgeries performed in the Netherlands over the past decade. Our hypothesis was that the introduction of the guideline may have contributed to a reduction in the number of surgeries for degenerative meniscus tears.

## Materials and methods

Registry-based data on arthroscopic procedures for meniscus tears used in the preparation of this observational study originated from the Dutch National Hospital Care Basic Registration (LBZ): a registry that is managed by the Dutch Hospital Data Foundation [23]. The Dutch Hospital Data Foundation, which was established by the Dutch Association of Hospitals (NVZ) and the Netherlands’ Federation of University Medical Centres (NFU), manages, maintains and monitors collections of hospital data and provides information on hospital care.

The National Hospital Care Basic Registration is a nationwide registry (covering data from all Dutch hospitals and university medical centres) of clinical-, administrative- and financial data from patients who have been hospitalised, received ambulatory surgery, or were treated in an outpatient setting. The registry includes: information on patient demographics; primary- and secondary diagnoses, in terms of International Classification of Diseases (ICD) codes; and surgical procedures performed.

Following Dutch privacy laws that ensure the anonymity of the provided data (i.e., not reducible to individual subjects or institutions), the Dutch Hospital Data delivered anonymous and aggregated data on subjects who had undergone meniscus surgery, as a primary- or secondary procedure (specific procedural codes for meniscus surgery: ZA code 038643/CVV code 5804X/CBV codes 338645X/338646X) from 2005, up to, and including, 2014. The data included: the type of hospital at which the surgery was performed (general hospital or university medical centre); the subject’s age (divided into 5-year age cohorts) and gender; and the patient’s primary diagnosis (coded according to ICD-9).

The numbers of inhabitants in the Netherlands of each registration year from 2005 to 2015 were obtained from the Statistics Netherlands [24]. Total numbers of inhabitants were retrieved, as well as numbers stratified for gender and age groups (0–20, 20–40, 40–65, 65–80, and 85 years and older) to calculate age- and gender-specific incidences of meniscal surgeries [24].

IRB approval was not required for this study, because it did not involve human subject research, and the data provided by the Dutch Hospital Data was anonymous and did not contain identifiers or codes linked to individuals.

### Statistical methods

Gender- and age-adjusted incidences were calculated to account for the ageing population over the successive registration years. For each registration year, data included the number of inhabitants on January 1, and number of subjects who had undergone meniscus surgery between January 1 and December 31. To account for the possible change in number of inhabitants during the registration year (from January 1 to December 31), we calculated mid-year population numbers. These were calculated as the average population number of the registration year and the following year. These mid-year population numbers were subsequently used to calculate the annual incidence rates of the meniscus surgeries per 100,000 people. Herein, we used population numbers stratified for gender and defined age groups to calculate gender- and age group-specific incidence numbers.

Poisson regression models were used to test the differences in incidences of meniscus surgeries performed in the Netherlands between 2005 and 2014. First, it was assessed if there was a difference in the incidences of meniscus surgeries performed between males and females, and between different age groups during 2005 to 2014. Second, it was assessed if the incidences changed over the successive registration years (i.e., registration years were modelled as a continuous covariate), and if incidences differed before- and after implementation of the national guideline in 2010 (i.e., incidences during 2004–2009 were compared to incidences during 2010–2014). Third, it was assessed if any change in incidences over the successive registration years differed for younger and older patients [for this purpose age groups were dichotomised in younger patients (<40 years) and middle-aged and older patients (>40 years), and successive registration years were modelled as a continuous covariate).

SPSS version 22.0 (SPSS, Chicago, IL) was used for statistical analyses. The significance level was set at *p* < 0.05.

## Results

**Fig. 1:**
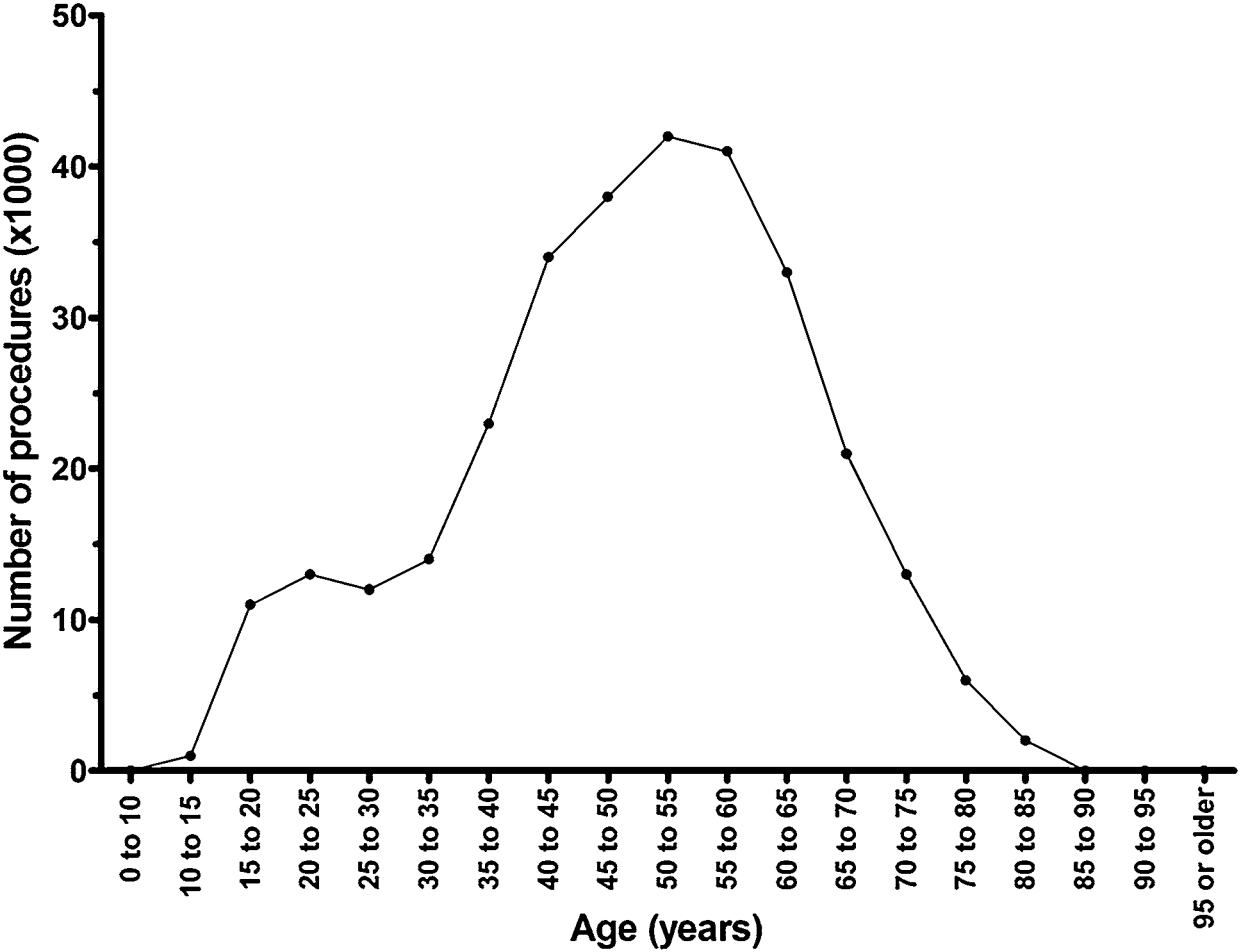
Cumulative numbers (thousands) of meniscal procedures performed between 2005 and 2014 plotted against the age groups (5-year cohorts)

From 2005 to 2014, a total of 304,307 meniscus surgeries were registered, of which the majority comprised of surgeries in middle-aged and older patients (Fig. 1). Of the total number of meniscus surgeries, 98% were performed in general hospitals and only 2% in university medical centres.

**Fig. 2:**
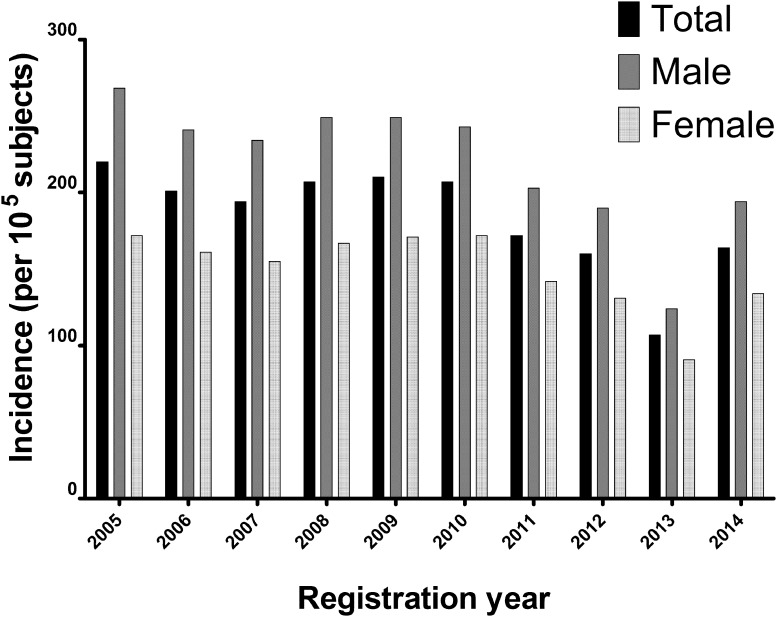
Between 2005 and 2014, the incidence of meniscus surgeries was higher for men than for women (*p* < 0.001). The incidence of meniscus surgeries decreased from 2005 to 2014 (*p* < 0.001). Evaluation of the incidences before and after the implementation of the Dutch national guideline in 2010 showed lower incidences after the introduction of the national guideline compared to before its implementation (*p* < 0.001)

The incidence of meniscus surgeries was higher for men than for women (*p* < 0.001), and highest in people aged 65–80 years old (*p* < 0.001) compared to the other age groups (Table 1). From 2005 to 2014, the incidence of meniscus surgeries decreased (*p* < 0.001). Evaluation of the incidences before- and after the implementation of the national guideline in 2010 demonstrated that incidence lessened after the introduction of the national guideline, compared to before its implementation (*p* < 0.001) (Fig. 2). The decrease in incidences over the registration year was different for the various age groups, in which the incidences for middle-aged and older (>40 years) patients decreased at a lower rate than the incidences in younger patients (<40 years) (*p* < 0.001).

**Table 1:** Number and incidences of meniscus procedures per year from 2004 up to and including 2014, stratified for gender and age

	Year										Total
	2005	2006	2007	2008	2009	2010	2011	2012	2013	2014	
Number of procedures											
Total, *n* (% of 10 year period)	35,823 (0.12)	32,797 (0.11)	31,769 (0.10)	34,034 (0.11)	34,583 (0.11)	34,312 (0.11)	28,654 (0.09)	26,843 (0.09)	17,972 (0.06)	27,520 (0.09)	304,307 (1.00)
According to gender, *n* (% of total registration year)											
Men	21,630 (0.60)	19,486 (0.59)	18,961 (0.60)	20,179 (0.59)	203,25 (0.59)	19,937 (0.58)	16,707 (0.58)	15,764 (0.59)	10,285 (0.57)	16,156 (0.59)	179,430 (0.59)
Women	14,193 (0.40)	13,311 (0.41)	12,808 (0.40)	13,855 (0.41)	14,258 (0.41)	14,375 (0.42)	11,947 (0.42)	11,079 (0.41)	7687 (0.43)	11,364 (0.41)	124,877 (0.41)
According to age, *n* (% of total registration year)											
0–20 years	1403 (0.04)	1232 (0.04)	1205 (0.04)	1339 (0.04)	1365 (0.04)	1340 (0.04)	1145 (0.04)	1122 (0.04)	734 (0.04)	1308 (0.05)	12,193 (0.04)
20–40 years	8428 (0.24)	7220 (0.22)	6730 (0.21)	6862 (0.20)	6642 (0.19)	6608 (0.19)	5238 (0.18)	5009 (0.19)	3167 (0.18)	5379 (0.20)	61,283 (0.20)
40–65 years	18,392 (0.51)	1,6727 (0.51)	16,130 (0.51)	17,329 (0.51)	17,850 (0.52)	17,410 (0.51)	14,620 (0.51)	13,763 (0.51)	9144 (0.51)	14,073 (0.51)	155,438 (0.51)
65–80 years	6593 (0.18)	6528 (0.20)	6656 (0.21)	7529 (0.22)	7747 (0.22)	7985 (0.23)	6849 (0.24)	6236 (0.23)	4402 (0.24)	6030 (0.22)	66,555 (0.22)
80 years and older	1007 (0.03)	1088 (0.03)	1048 (0.03)	975 (0.03)	979 (0.03	969 (0.03)	802 (0.03)	713 (0.03)	525 (0.03)	730 (0.03)	8836 (0.03)
Incidences (per 10^5^ persons/year)											
Total^a^	220	201	194	207	210	207	172	160	107	164	
According to gender^b^											
Men	268	241	234	249	249	243	203	190	124	194	
Women	172	161	155	167	171	172	142	131	91	134	
According to age^c^											
0–20 years	35	31	30	34	35	34	29	29	19	34	
20–40 years	189	164	156	161	157	158	126	121	77	131	
40–65 years	331	297	282	300	305	294	244	230	153	237	
65–80 years	384	374	377	418	421	422	355	307	207	274	
80 years and older	176	185	174	158	155	150	120	104	75	102	

^a^Annual incidence rates calculated as number of meniscal procedures performed per 100,000 Dutch inhabitants, from 2005 to 2014 the incidence of meniscus surgeries decreased (*p* < 0.001), and the incidences were lower after the introduction of the national guideline compared to before its implementation (*p* < 0.001)

^b^Gender specific incidence numbers, per 100,000 male/females, the incidence of meniscus surgeries was higher for men than for women (*p* < 0.001)

^c^Age corrected incidence numbers, per 100,000 Dutch inhabitants per age cohort, the incidence of meniscus surgeries was highest in persons aged 65–80 years (*p* < 0.001) compared to other age groups

A sensitivity analysis, in which the year 2013 was omitted from the analysis, demonstrated similar results. Six diagnoses represented 99% of all the primary diagnoses, of which three were nonspecific (‘other and unspecified disorders of joint’, ‘other disorders of bone and cartilage’, and ‘other derangement of joint’). The three most common specific primary diagnoses were: old meniscus tear (i.e., derangement of the meniscus due to an old tear or injury); osteoarthritis; and acute/current meniscus tear. An old meniscus tear was the predominant diagnosis over all registration years and accounted for 93% of the registered procedures (Table 2).

**Table 2:** Most common primary diagnosis for patients undergoing meniscus procedures over 2005–2014 in The Netherlands

	Registration year										Total
	2005	2006	2007	2008	2009	2010	2011	2012	2013	2014	
Number of meniscal procedures											
Total, *n*	35,823	32,797	31,769	34,034	34,583	34,312	28,654	26,843	17,972	27,520	304,307
According to primary diagnosis^a^, *n* (% of total procedures in registration year)											
Old meniscus tear^b^	33,811 (0.94)	30,777 (0.94)	29,940 (0.94)	32,265 (0.95)	32,694 (0.95)	32,246 (0.94)	26,503 (0.92)	24,622 (0.92)	16,749 (0.93)	24,176 (0.88)	283,783 (0.93)
Osteoarthritis^c^	475 (0.01)	426 (0.01)	431 (0.01)	436 (0.01)	453 (0.01)	394 (0.01)	536 (0.02)	272 (0.01)	169 (0.01)	368 (0.01)	3960 (0.01)
Acute meniscus tear^d^	125 (0.00)	90 (0.00)	58 (0.00)	147 (0.00)	77 (0.00)	116 (0.00)	153 (0.01)	130 (0.00)	114 (0.01)	508 (0.02)	1518 (0.00)
Other^e^	1136 (0.03)	1102 (0.03)	994 (0.03)	812 (0.02)	1098 (0.03)	1278 (0.04)	1102 (0.04)	1387 (0.05)	713 (0.04)	1191 (0.04)	10,813 (0.04)
Total	35,547 (0.99)	32,395 (0.99)	31,423 (0.99)	33,660 (0.99)	34,322 (0.99)	34,034 (0.99)	28,294 (0.99)	26,411 (0.98)	17,745 (0.99)	26,243 (0.95)	300,074 (0.99)

^a^Most common primary diagnose for patients undergoing meniscus surgery according to ICD-9

^b^Old meniscus tear (ICD-9 code 717 Internal derangement of knee, i.e., derangement of the meniscus due to old tear or injury),

^c^Osteoarthritis (ICD-9 code 715 Osteoarthrosis and allied disorders)

^d^Acute/current meniscus tear (ICD-9 code 836 dislocation of knee)

^e^Other unspecified disorders/derangement *ICD codes 719 Other and unspecified disorders of joint, 733 Other disorders of bone and cartilage, and 718 Other derangement of joint

## Discussion

The most important finding of this study was that the incidences of meniscus surgeries decreased from 2005 to 2014. This decrease was observed in all age groups, although the decrease in incidences was more pronounced for younger patients (aged less than 40 years) compared to middle-aged and older patients (aged 40 years and older). Moreover, the majority of meniscus surgeries were performed on middle-aged and older patients (aged 40 years and older).

In contrast to our results, several studies have demonstrated increased incidences of surgeries for degenerative meniscus tears in different countries. Thorlund et al. reported that the incidence of meniscus surgery had doubled from 2000 to 2011 in Denmark, including a striking threefold increase in subjects aged 55 years and older [21]. This increase was demonstrated to be particularly attributable to a rise in procedural incidences in the private sector [25]. Lazic et al. reported a more than twofold increase in arthroscopic meniscal resections from 2000 to 2012 in the UK, with particular high increases in subjects aged 60–74 years (which almost quadrupled) [26]. Matilla et al. reported an increased incidence of surgery for degenerative meniscal tears in Finland from 1997 to 2007, but not in Sweden [27]. Kim et al. reported an increase in arthroscopic procedures for treatment of meniscal tears, especially among middle-aged patients between 1996 and 2006 in the United States [28].

Different trends in meniscus surgery incidences between various countries are probably not solely explained by differing incidences of knee degeneration and traumatic injuries. The implementation of a nationwide guideline in the Netherlands may, in part, be responsible for the absence of an increase in incidences in meniscus surgeries, as observed in the other countries. However, other reasons may also have contributed to the observed differences. Differences in physician beliefs about procedural effectiveness, changes in surgical coding (e.g. many cases that would have previously been coded for knee osteoarthritis may be coded more recently as meniscus tears, because the use of arthroscopy for knee osteoarthritis is no longer reimbursed by several insurance companies, and many knees with osteoarthritis also demonstrate meniscal tears [28, 29]), financial incentives, and differing rates of dissemination, acceptance, and implementation of clinical evidence in the practice of arthroscopic surgery can be put forward as possible contributors to the observed differences.

The decrease in incidence of meniscus surgeries observed in the current study may in part be explained by the introduction of a guideline for knee arthroscopy by the Netherlands Orthopaedic Association in 2010 [22]. This guideline succeeded the consensus indication for arthroscopy in acute knee injuries from 1998 [30]. The 2010 guideline introduced the use of MRI in the diagnostic process of meniscus tears in younger patients. Using the MRI in the diagnostic process may have led to a decrease in meniscus surgeries by providing additional diagnostic information in patients with non-specific symptoms. Alongside the recommended use of MRI, the 2010 guideline also advises surgeons to apply caution to the surgical treatment of degenerative meniscus tears, and recommends non-operative management of degenerative meniscus tears without mechanical obstructions [22]. These recommendations may have been responsible for the observed reduction in meniscus surgeries. However, remarkably, the high incidence in middle-aged and older patients after 2010 remains, in contrast to the growing evidence that questions the effectiveness of the procedure. The lack of a more pronounced decrease in procedural incidences in patients aged 40 years and older demonstrates a delay in dissemination, acceptance and implementation of clinical evidence in the practice of arthroscopic surgery in the Netherlands.

For this registry-based study, coverage and the validity are potential limitations. Although Dutch hospitals are not legally required to participate in the National Hospital Care Basic Registration, all hospitals affiliated with the Dutch Association of Hospitals (NVZ) and the Netherlands Federation of University Medical Centres (NFU) have a statutory obligation to participate in the registration. Therefore, virtually all Dutch hospitals participate in the Registration [23]. Alongside this, the validity of the registration of demographics, diagnoses, and procedures has been demonstrated to be good (correct registration of 99%, 87% and 92%, respectively) [31]. We assumed that validity of registered demographics, diagnoses and procedures was comparable between 2005 and 2014. Completeness of registrations is another potential limitation. The year 2013 stands out due to the disproportionately low number of registered procedures compared to other years. Whereas absolute numbers are lower, proportions of procedures performed on men and women and for defined age groups did not differ from other years. Lower numbers of registered procedures can be related to a nationwide change in the registry platform. The year 2013 was the last during which registry data was accepted via the old platform, and from January 1st 2014 onwards, the new registry definitively replaced the former platform. A change in infrastructure for management of registry data at hospital-level in 2013 may have led to incomplete submission of data to the registry in that year. Disregarding 2013, we assumed that completeness of the registry was comparable over the registration years. Sensitivity analyses demonstrated that omitting 2013 from the analyses did not influence the interpretation of the results.

Another limitation is that the ICD codes, as well as the procedural codes for meniscus surgery, did not discern between meniscectomy versus suture repair versus root repair, hence, all meniscus procedures were included.

The observation that, despite accumulating evidence that questions the rationalisation and effectiveness of the treatment, meniscus surgery is still widely performed in the treatment of degenerative meniscus tears demonstrates a delay in the dissemination, acceptance, and implementation of clinical evidence in the practice of arthroscopic surgery in the Netherlands. Moreover, it signals the need to examine why the numbers of meniscus surgeries in middle-aged and older patients remain disproportionately high. The identification of these reasons (i.e. barriers) may subsequently lead to the development of tailored implementation strategies for updated guidelines.

## Conclusion

In conclusion, the implementation of a nationwide guideline for arthroscopic procedures for meniscus tears may have contributed to a decrease in incidences of meniscus surgeries. However, despite accumulating evidence that questions the rationalisation and effectiveness of the treatment, meniscus surgery is still widely performed in the treatment of degenerative meniscus tears.
